# Object Recognition in High-Resolution Indoor THz SAR Mapped Environment

**DOI:** 10.3390/s22103762

**Published:** 2022-05-15

**Authors:** Aman Batra, Fawad Sheikh, Maher Khaliel, Michael Wiemeler, Diana Göhringer, Thomas Kaiser

**Affiliations:** 1 Institute of Digital Signal Processing, Universität Duisburg-Essen, 47057 Duisburg, Germany; fawad.sheikh@uni-due.de (F.S.); maher.ahmed@uni-due.de (M.K.); michael.wiemeler@uni-due.de (M.W.); thomas.kaiser@uni-due.de (T.K.); 2 Benha Faculty of Engineering, Benha University, Benha 13511, Egypt; 3 Chair of Adaptive Dynamic Systems, Technische Universität Dresden, 01069 Dresden, Germany; diana.goehringer@tu-dresden.de

**Keywords:** synthetic aperture radar, terahertz imaging, indoor imaging, high-resolution indoor environment, indoor object recognition

## Abstract

Synthetic aperture radar (SAR) at the terahertz (THz) spectrum has emerging short-range applications. In comparison to the microwave spectrum, the THz spectrum is limited in propagation range but benefits from high spatial resolution. The THz SAR is of significant interest for several applications which necessitate the mapping of indoor environments to support various endeavors such as rescue missions, map-assisted wireless communications, and household robotics. This paper addresses the augmentation of the high-resolution indoor mapped environment for object recognition, which includes detection, localization, and classification. Indoor object recognition is currently dominated by the usage of optical and infrared (IR) systems. However, it is not widely explored by radar technologies due to the limited spatial resolution at the most commonly used microwave frequencies. However, the THz spectrum provides a new paradigm of possible adaptation of object recognition in the radar domain by providing image quality in good compliance to optical/IR systems. In this paper, a multi-object indoor environment is foremost mapped at the THz spectrum ranging from 325 to 500 GHz in order to investigate the imaging in highly scattered environments and accordingly create a foundation for detection, localization, and classification. Furthermore, the extraction and clustering of features of the mapped environment are conducted for object detection and localization. Finally, the classification of detected objects is addressed with a supervised machine learning-based support vector machine (SVM) model.

## 1. Introduction

Object recognition is the core of many emerging applications such as autonomous vehicles, household robotics, face-id smartphones, and security [1]. The object recognition technique involves a set of collective computer vision tasks for the analysis of objects in a digital image. The tasks could be the detection, localization, and classification of objects, which are a subset of object recognition. This technique primarily belongs to the field of computer vision, where mainly optical sensors such as an RGB camera and light detection and ranging (LiDAR) are employed [1,2,3]. Therefore, the optical sensors dominate the field, owing to the very high spatial resolution available in the range of μm [4] at the optical spectrum. However, the optical sensors are limited in terms of penetration depth and highly dependent on environmental conditions such as daylight and weather. In this regard, radar imaging prevails.

In the 1950s, synthetic aperture radar (SAR) was developed as an alternative to optical imaging systems for defense applications [5]. SAR is a remote sensing technique and is well known for 2D and 3D imaging. In this technique, the radar sensors are mounted on a mobile platform, and a large aperture is synthesized to acquire high angular or cross-range resolution. Presently, it is used in a wide variety of applications such as topographic imaging, meteorology, surveillance, land and vegetation structure observation, and underground resource exploration [5,6]. State-of-the-art SAR applications are mainly based on the microwave spectrum. This spectrum benefits from a large penetration depth and sensing range, but the available spatial resolution is limited. This limitation hinders the adaptation of vision-based object recognition techniques to radar imaging or sensing. In SAR technology, the spatial resolution is classified into range and cross-range resolution, which are directly proportional to frequency, bandwidth, and antenna dimensions.

Recently, the terahertz (THz) spectrum has attracted significant interest [7]. The novel extension of SAR to the THz spectrum enables a new era of SAR applications. Due to the available large bandwidth, smaller wavelengths, and compact antennas at the THz spectrum, sub-mm spatial resolution is achievable [8]. In comparison to the microwave spectrum, the THz spectrum is limited in sensing range due to higher atmospheric attenuation, free space path loss, and lower transmit power [9,10]. Despite the previously mentioned limitations, the THz SAR sensing is suitable for short-range applications, especially in indoor environments. An application example is the indoor rescue mission for emergency scenarios, where multiple sensors are employed. In this case, the optical and infrared (IR) sensors might not provide any useful information. However, the THz SAR sensors can generate a high-resolution map of the environment. The map could be extended for the autonomous detection, localization, and classification of objects such as humans and electrical wires, which will be extremely dangerous in such situations.

Complementary to the indoor THz SAR applications, many novel THz SAR testbeds are proposed in the literature in areas such as the automobile [11], non-destructive testing [12], and security [13]. The object recognition serving security purposes is presented in [14,15,16,17]. In [14,15,16], imaging is conducted with a photonics system, where the signal to noise ratio is limited and also images have lower contrast. Active imaging with the frequency modulated continuous wave (FMCW) radar system is employed in [17]. The prime focus in [14,15,16,17] is the recognition of objects beneath the clothes targeting security applications. Recently, object analysis at the THz spectrum from the NDT perspective is presented in [12]. In the automotive field, object recognition based on radar-cross-section (RCS) sensing is of significant interest and powered by the commercially available radar chips at 77 GHz [18]. In this paper, indoor environment profiling is in the foreground.

The paper’s contribution is foremost to generate a high-resolution 3D indoor environmental map, where the environment is enriched with multiple objects. The map is generated at the THz spectrum of 325–500 GHz with a vector network analyzer (VNA)-based testbed. The indoor objects are considered in a group of 2 and 4. Concealed and hidden object scenarios are also considered to validate the objectives of object recognition in both free-space and concealed cases. The high-resolution environment map is processed for object recognition (detection, localization, and classification). For object detection, speeded up robust features (SURF) [19] are extracted, and features are clustered in groups based on the density-based spatial clustering of applications with noise (DBSCAN) algorithm [20,21]. Due to the 3D mapped environment, 3D positions of the detected objects can be estimated with an accuracy in the range of mm. Finally, the classification of detected objects is addressed using machine learning techniques. The THz training dataset is scarce, and especially for indoor objects, no public-domain dataset is available. Hence, a dataset is developed and a supervised machine learning-based support vector machine (SVM) model is implemented. Lastly, the model robustness is also evaluated. It is worth to mentioning that some of the work presented in this paper belongs to the principal author’s dissertation [22].

The remainder of the paper is organized as follows. Section 2 explains the SAR signal processing. In Section 3, a multi-object environments mapping with the THz SAR technique is demonstrated. Section 4 addresses object detection and localization. The classification of the detected objects is presented in Section 5. Lastly, the concluding remarks and outlook perspectives are presented in Section 6.

## 2. SAR Theoretical Model

This section explains the 3D SAR signal processing in consideration of monostatic configuration, where the transmitter and receiver are collocated and driven by the same reference oscillator. The SAR principle could be explained as a radar sensors or transceiver system, which is mounted on a mobile platform and synthesizes a large antenna aperture in order to acquire high-angular resolution. Here, the aperture is synthesized by following a certain trajectory along cross-range directions. During the trajectory, electromagnetic (EM) waves are transmitted toward the target along the range direction and echoes are recorded, which form the raw data. For target analysis, the raw data are processed with an image reconstruction algorithm. In the following subsection, the mathematical model of raw data acquisition considering point targets and image reconstruction is presented.

### 2.1. Raw Data

**Figure 1:**
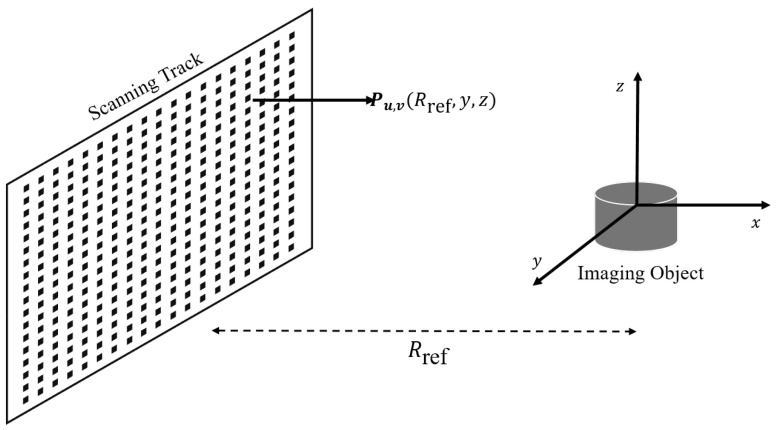
Three-dimensional (3D) imaging geometry (reproduced from [22]).

In this paper, a planar aperture configuration is applied, where the 3D imaging is acquired by implementing a 2D trajectory along the azimuth and elevation directions. Figure 1, which is reproduced from [22], presents the 3D imaging geometry, where the *x*-, *y*- and *z*-axis represent range, azimuth, and elevation directions, respectively. The transceiver located at position **P**${}_{u,v}$ transmits and records the backscattered EM waves at each aperture position. The parameters (u,v) are the respective azimuth and elevation coordinates based on the presented geometry, where u∈(1,U), v∈(1,V), whereas *U* and *V* are the total number of aperture positions along the *u* or *y*-axis and *v* or *z*-axis, respectively. The total number of aperture positions in the 2D scanning track can be given by N=U×V.

Let us consider that the transceiver at position **P**${}_{u,v}$ transmits a signal p(t) which could be of any waveform such as Gaussian or chirp. The received signal, which is a time-delayed version of the transmitted signal and backscatted by *K* scatterers, could be expressed by (1)

(1) $$s\left(t,P_{u,v}\right)=\sum_{k=1}^{K}A_{k}p\left(t-t_{d,k}\right),$$

where $t_{d,k}=\frac{2R_{k}}{c}$ is the round-trip delay, and $R_{k}$ is the slant range between the *k*th scatterer located at position $\left(x_{k},y_{k},z_{k}\right)$ and transceiver [5]. Furthermore, $A_{k}$ is the amplitude of the reflectivity from the *k*th scatterer, which is assciated with the target RCS. At each aperture position, the backscattered signal $s(t,P_{u,v})$ is recorded and forms the raw data. In Figure 1, the reference position is defined as $R_{\mathrm{ref}}$, and the coordinates of the scatterer position and transceiver could be positive or negative from the considered reference center of origin.

### 2.2. Image Reconstruction

The raw data are processed further with time- or frequency-domain image reconstruction algorithms. For example, *Backprojection Algorithm* (BPA) is a time-domain algorithm and *Range Doppler* and *Omega-K* are frequency-domain algorithms. In this work, BPA is used due to its simplicity. Although BPA requires more computational power, it has inherent massive parallelism to accelerate image reconstruction for real-time applications [23]. In addition, the BPA algorithm is less sensitive to motion errors [24].

For a VNA-based testbed, the raw data are gathered in the frequency domain and zero-padded before the transformation to time-domain defined as $s_{z}\left(t,P_{u,v}\right)$ using inverse Fourier transform. The zero-padding is performed for increasing the resolution in the time-domain. Based on BPA, in the 3D image reconstructed grid I, voxel value at location $\left(x_{i},y_{j},z_{k}\right)$ is given by (2)

(2) $$I\left(x_{i},y_{j},z_{k}\right)=\sum_{U}\sum_{V}s_{z}\left(t_{d,ijk},P_{u,v}\right)\exp\left(j2\pi f_{\min}t_{d,ijk}\right)$$

where $t_{d,ijk}$ is the round-trip delay between the scatterer at the voxel position $\left(x_{i},y_{j},z_{k}\right)$ in I and transceiver assumed at position $P_{u,v}$, and $f_{\min}$ is the minimum frequency of the zero-padded signal. At the THz spectrum, a high-resolution 3D image is generated with Equation (2).

The resolution defines the minimum resolvable distance between two scatterers. In the SAR technique, the spatial resolution is defined along the range and cross-range directions. The range resolution is proportional to bandwidth $B_{w}$ and given by (3)

(3) $$r_{x}=\frac{c}{2B_{w}}.$$

For the presented imaging geometry in Figure 1, the cross-range resolution is proportional to the antenna half power beamwidth and represented by (4)

(4) $$r_{y}=r_{z}=\frac{\lambda R_{\mathrm{ref}}}{2L_{s}},$$

where λ is the center frequency and $L_{s}$ is the synthetic aperture length. For a trajectory of length $L_{s}=\lambda R_{\mathrm{ref}}/L_{a}$, where $L_{a}$ is the antenna diameter, the cross-range resolution could be approximated as $L_{a}/2$ [25]. It could also be defined as the maximum achievable cross-range resolution if the previously mentioned condition of $L_{s}$ is fulfilled.

To summarize, the large available bandwidth in addition to small wavelengths and compact antennas at the THz spectrum results in high spatial resolution. For example, $B_{w}=175\;\mathrm{GHz}$ and $L_{a}=1.93\;\mathrm{mm}$ provide a range resolution of 0.86mm and cross-range resolution of 0.965mm.

## 3. Multi-Object Environment Mapping

This section addresses 3D multi-object environment mapping. The objective is firstly to demonstrate a THz SAR-based 3D multi-object mapping, especially with indoor objects in a cluttered environment. The second objective is to generate a high-resolution map that can be elaborated for object detection, localization, and classification. The following subsections present the measurement setup and the acquired 3D SAR images.

### 3.1. Measurement Setup

**Figure 2:**
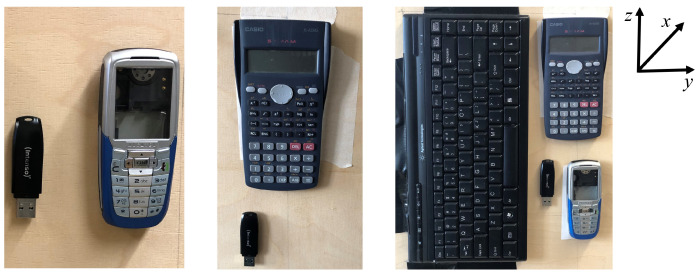
Photograph of objects (reproduced from [22]) in (**left**) case I, (**middle**) case II, and (**right**) case III.

**Figure 3:**
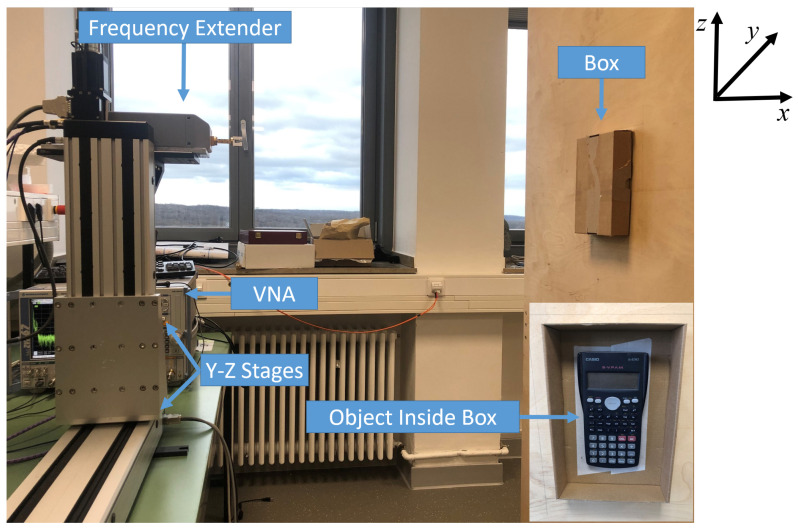
Measurement setup of case IV and the indoor environment.

For realization of the above described objectives, four cases are presented. In cases I and II, two objects are considered, which are displaced horizontally and vertically as shown in Figure 2 (left) and (middle). Figure 2 is reproduced from [22]. Case III combines both previous cases with four objects scattered horizontally and vertically, as shown in Figure 2 (right). Here, the considered objects are a keyboard, calculator, mobile phone, and universal serial bus (USB) stick, as shown in Figure 2. Furthermore, in case IV, the THz sensing of concealed and hidden objects is considered. In this case, an object (calculator), is placed in a cardboard box, as shown in Figure 3.

The measurement setup is shown in Figure 3, where a VNA coupled with a frequency extender is employed as a THz radar transceiver. A rectangular horn antenna with $L_{a}=1.93\;\mathrm{mm}$ and an average antenna gain of ∼25 dB at the frequencies of interest is connected to the extender waveguide flange. The VNA operates in the frequency range of 10MHz to 67GHz. The low-frequency signal from the VNA is up-converted by the frequency extender into the desired THz spectrum of 325–500 GHz. To form the synthetic aperture by implementing a 2D trajectory, the frequency extender is mounted on the Y-Z stages. At each position $P_{u,v}$, $S_{11}$ reflection coefficients are captured, which forms a stop-and-go approximation system. It is also worth mentioning that the measurements are performed without a prior wireless channel subtraction. Hence, the measurements are recorded in the presence of noise or clutter. The noise in this scenario is the unwanted reflections from all the objects existing in the environment except the imaging object—for example, a wooden block on which the box is mounted, as shown in Figure 3. However, most of the reflections from non-interested objects will not be focused by applying a time-gating window. The multi-path clutter/noise will be non-coherently superimposed due to the recorded measurements at different aperture positions.

The measurement parameters are shown in Table 1 along with the distribution of the objects in all four cases. The aperture lengths are considering based on the reference range and mapping area. In case 3 only, a set of two $L_{s,y}$ is considered to optimize the measurement time by not measuring the empty area below the mobile phone. For $L_{s,z}\in \left(1,233\right)\;\mathrm{mm}$, it is 300mm, and it is 270mm for $L_{s,z}\in \left(234,284\right)\;\mathrm{mm}$.

### 3.2. 3D SAR Image

The generated raw data are processed with BPA, and 3D SAR image matrix I is generated using Equation (2). For cases I–III, the raw or measurement data produced in [22] are used for SAR image reconstruction.

**Figure 4:**
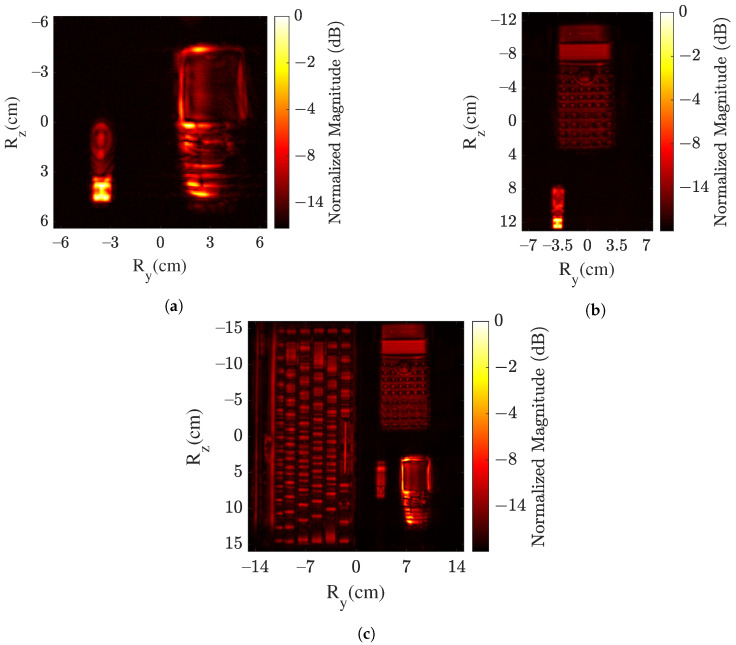
SAR image of object’s surface using MIP method in (**a**) case I, (**b**) case II and (**c**) case III.

Visualization of the 3D images in a 2D plane is also one of the concerns. Therefore, the maximum intensity projection (MIP) method is used for visualization. Based on this method, the voxels with maximum intensity in each range layer of I are projected. The projected images for cases I–III are shown in Figure 4. In the presented SAR images, a nice visualization of the object’s surface in a 2D plane is in the foreground for the purpose of object recognition. The objects are projected in close approximation to the $R_{\mathrm{ref}}$. Another method of visualization is a scattered plot, which provides the information in context of the position in 3D space, but the object’s shape is challenging to observe. Hence, the MIP is considered for better visualization.

For case I, both objects are well mapped, as shown in Figure 4a. The keys of the mobile phone are clearly visible. The right side of the mobile phone is intentionally left unfocused in this imaging case. The core target for object detection and classification is that the object should be detected and classified even if the image consists of artifacts. Moreover, in case I, the USB stick shape is also well observable in Figure 4a. The metallic component and the plastic material of the USB stick body are discernible. Moreover, the small rectangular holes in the metallic component are observable. Figure 4b shows the SAR image for case II. Similar to the previous case, both objects are well mapped, and the complete shape of the calculator is observable. Furthermore, Figure 4c presents the resulting SAR image for case III with four objects. In this case, a well-focused SAR image showing all the four objects is generated. Smaller structures of the objects, such as keys of the keyboard/mobile/calculator, are well displayed.

**Figure 5:**
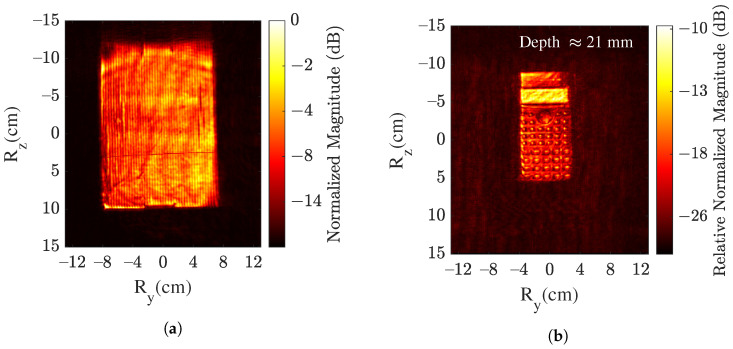
SAR image using MIP method in case IV of projected (**a**) box’s surface and (**b**) object’s surface.

The resulting SAR images for case IV are presented in Figure 5. Firstly, the box surface image is shown in Figure 5a. For sensing the object inside the box, the depth layer needs to be analyzed. It is analyzed by truncating the image plane along the range with a time-gating window. At a depth layer of 21mm, the imaging object (calculator) is extracted and shown in Figure 5b. A high-resolution image of the calculator is obtained, which is of similar accuracy as in cases II and III. The magnitude is relatively normalized to the reflection from the box surface. It can be seen that the box surface results in an attenuation of ∼10 dB.

To summarize, in all the four cases, high-resolution SAR images are generated, which are in good compliance to the optical sensors such as RGB cameras. Especially, in case IV, the optical sensors do not provide any information. Moreover, in addition to the object surface image in all the cases, the inside components such as the microcontroller of the object’s body can be precisely mapped. In the following section, the acquired high-resolution SAR images will be further evaluated for object detection and localization.

## 4. Object Detection and Localization

This section addresses the detection and localization of objects in a high-resolution SAR image. For the presented multi-object THz SAR imaging (cases I–IV), the objective is to acquire the identification in terms of the number of mapped objects and their respective positions in a 3D environment. The localization information is provided in reference to the transceiver position. The positions based on the environment geometry can also be extracted if the transceiver position is known in reference to the environment geometry. For example, let us consider indoor THz SAR sensing assisted with an indoor localization system presented in [26]. The localization system tracks the SAR trajectory and provides the transceiver position ($p_{x},p_{y},p_{z}$) in reference to the indoor room geometry of a certain dimension. Based on the object localization approach in this work, the object position $\left(t_{x},t_{y},t_{z}\right)$ can be obtained from the 3D SAR image. To be noted, the positions $\left(t_{x},t_{y},t_{z}\right)$ can be positive or negative based on the reference position considered for acquiring SAR geometry. With the fusion of transceiver (in reference to room geometry) and object positions (in reference to the mapped environment), the actual positions of the object in the room can be given by (5)

(5) $$Z_{ob}\left(x,y,z\right)=\left(p_{x}+t_{x},p_{y}+t_{y},p_{z}+t_{z}\right)$$

**Figure 6:**
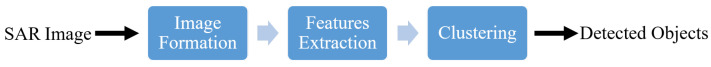
Workflow of the object detection.

Hence, if the objects are detected in the SAR image, their respective positions could be provided. The geometric properties such as the height, length, width, and thickness of the detected objects could be acquired as well. Figure 6 presents the workflow for object detection. The method comprises three stages: image formation, features extraction, and clustering. The description of these stages is explained in the following subsections.

### 4.1. Image Formation

In this block, the input is a high-resolution SAR image for cases I–IV presented in Section 3. The input image is generated in a grayscale color scheme. The scheme is selected as the SAR image pixels do not represent RGB values such as the image generated with optical systems. In addition, to be noted, the input image is in the portable network graphics (PNG) format. Any other graphics format such as joint photographic experts group (JPEG) can also be considered.

In the image formation block or module, the grayscale SAR image is processed to reduce the clutter and artifacts. For object detection, the boundaries of the object are of significant importance in comparison to object shape, components, or parts. In this scope, the standard approaches are based on edge detectors such as Canny detector [27] and combined corner/edge detector, for example, Harris detector [28]. However, these approaches are challenging in the field of radar imaging as the SAR image does not have sharp boundaries in comparison to optical images. Therefore, in view of artifacts/clutter removal and focusing on object boundaries, the image is suppressed based on a certain threshold. The grayscale image consists of values between 0 and 255, where black color is represented by 0, and 255 represents a white color. Hence, the SAR image pixel intensities representing the EM field magnitude are normalized in the grayscale range. A threshold of 1dB is selected, which defines the pixel intensity below 1dB of the normalized maximum intensity (white color) as a minimum gray-scale value (black color). The concept of threshold parameter selection can be explained with the noise floor or dynamic range of the presented VNA-based testbed.

With a VNA-based testbed, the noise floor or level is related to the intermediate frequency (IF) $B_{w}$. For example, the noise floor at different IF $B_{w}$ for a similar VNA-based testbed in the spectrum range of 220–330GHz is presented in [29]. In this work, IF $B_{w}$ is 10KHz, where the noise floor is around −85dB. In the normalized grayscale SAR image, the maximum intensity is represented by this noise floor. Based on the considered threshold, every EM wave reflection from the environment with a magnitude of 1dB above the noise floor is considered in the threshold-based image formation.

**Figure 7:**
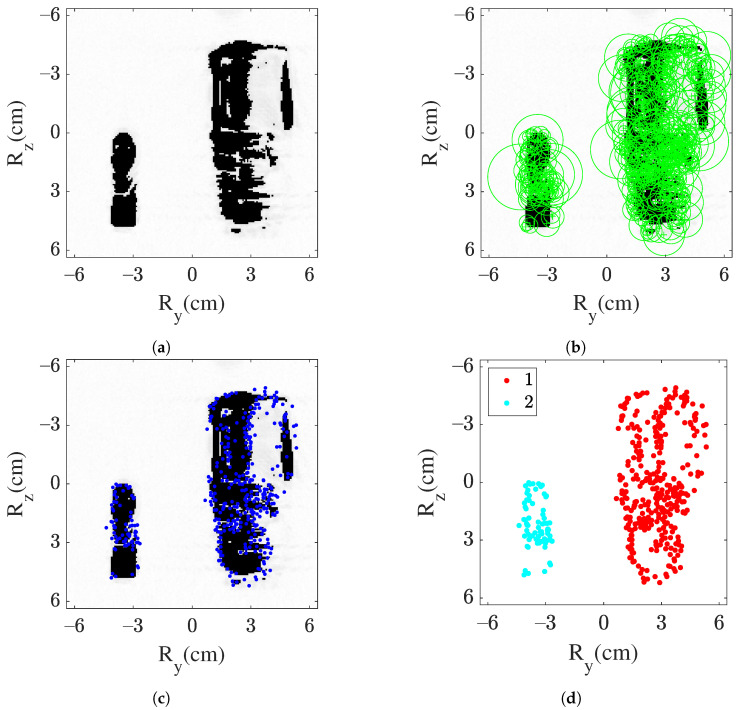
Output of object detection workflow for case I, (**a**) threshold image, (**b**) extracted features positions, (**c**) key points position, and (**d**) key points cluster grouping.

Following this approach of image formation, the parts of the object even with a lower backscattering coefficient are considered. It is beneficial in forming the boundaries of the object in the proposed method of object detection. Considering a case I SAR image as an input image, the output of the image formation block is shown in Figure 7a. In the resultant image, it can be seen that only the continuous shape along with boundaries is in focus.

### 4.2. Features Extraction

The next module after image formation is the extraction of interest points and descriptors from the image. There are many algorithms available for features extraction such as scale-invariant feature transform (SIFT) [30] and SURF [19]. In this work, SURF is employed as it is invariant to the scale, color, and geometric variations. The SURF relies on the integral image that can be computed and evaluated faster. The acceleration is essential for real-time applications. The SURF algorithm consists of a detector and descriptor. The detector is based on the Hessian matrix for finding the key or interest points, where the Hessian matrix elements are given by the convolution of image pixel position and the Gaussian second-order partial derivative. In this algorithm, the descriptor is based on the Haar wavelet response. A detailed explanation of the algorithm is available in [19].

In case I, Figure 7a is input to the feature extraction module. As the output of this module, Figure 7b,c show the positions of the extracted feature’s key points, respectively. Most of the extracted features and key points are within the boundaries of the objects.

### 4.3. Clustering

The next module after the feature extraction is clustering of the key points. Two widely used clustering algorithms are *k*-means [31] and DBSCAN [20,21]. In *k*-means, the key points are grouped into *k* clusters, where the *k* needs to be defined for clustering. Hence, this algorithm can not be applied in this work, where the focus is on autonomous object detection. A priori knowledge of *k* is not available, as the task is to obtain the number of clusters, which relatively defines the number of objects. On the other hand, the DBSCAN algorithm clusters the key points based on the density. It basically forms the clusters of a dense region without any prior knowledge of the number of clusters. Hence, the DBSCAN methodology is applied in this work.

In this methodology, the clusters are formed by identifying the number of minimum neighboring points within a specified radius. The radius $\epsilon_{r}$ is defined as the maximum distance between two key points that can be mapped to the same cluster. The key points or data points identified by the DBSCAN algorithm are categorized into the core, border, and noise. The core points are those which fall within the $\epsilon_{r}$, whereas the border points are defined as the points positioned on the edge of $\epsilon_{r}$. Lastly, the noise is defined as points which neither fit the core nor border points. Hence, the key point clusters are obtained with core and border points. The noise points will simply be discarded. In this work, for clustering, 10 minimum points are selected. Based on the size of the considered object and acquired high-resolution SAR images, it is expected that at least 10 key points are obtained for each object under the feature extraction module. The $\epsilon_{r}$ parameter associated with the distance of 1.5 cm is selected. This implies that the objects can be clustered successfully if the objects are at least separated by a distance of 1.5 cm and a minimum of 10 key points are available for each object. Moreover, the parameters can be adapted based on the sensing environment.

To summarize, extracted key points using the features extraction module are provided as an input to the clustering module. As an output, the clusters of key points are obtained as shown in Figure 7d for case I. Here, two clusters are obtained and hence validate the presented module.

### 4.4. Detected Objects

**Figure 8:**
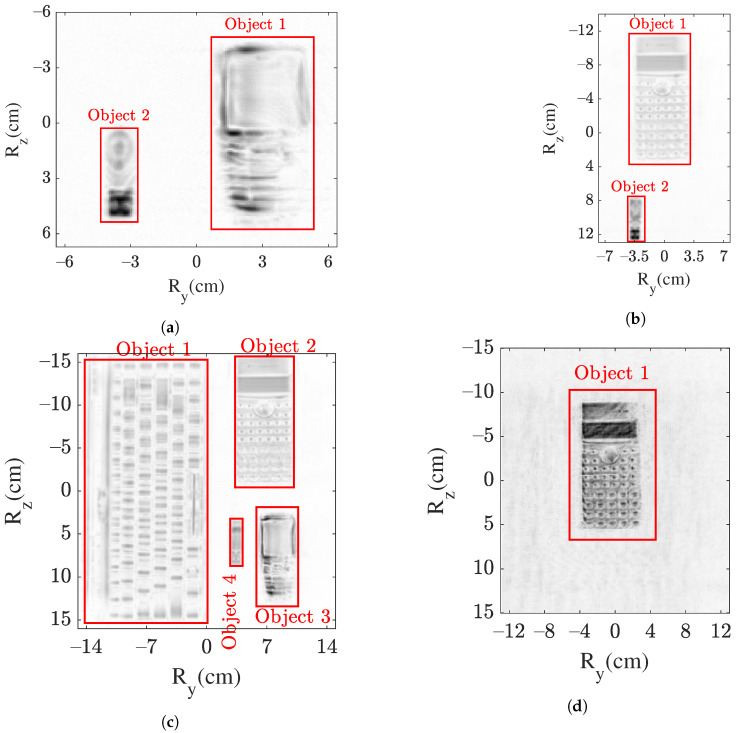
Detection of objects in an SAR image marked in a red rectangular window: (**a**) two in case I, (**b**) two in case II, (**c**) four in case III, and (**d**) one in case IV.

Based on the above-described modules, the detection of objects is obtained. The workflow is implemented in MATLAB. For case I, the detected objects are marked in the SAR image shown in Figure 8a. In case I, both of the objects are detected, and their respective locations ($t_{x},t_{y},t_{z}$) can be extracted. The coordinates $t_{y}$ and $t_{z}$ can be directly obtained from the center of the object’s cluster or the center of the rectangular window with red borders shown in Figure 8a. For example, for object 1, $t_{y}$ and $t_{z}$ are 3cm and 1cm, respectively. Similarly, for object 2, $t_{y}=$ −3.5cm and $t_{z}=$ 3cm. The coordinate $t_{x}$ can be obtained directly from the volumetric analysis where the generated 3D SAR image matrix *I* from Section 3 based on Equation (2) is used.

Similarly, for the other cases II–IV, the objects are detected based on the presented workflow. In cases II and IV, the parameter $\epsilon_{r}$ is adapted in accordance to meet the distance of 1.5cm due to the different imaging grid sizes and pixel dimensions. In case III, due to the large number of objects, they are in closer proximity compared to other cases. Therefore, a shorter distance of 1cm is considered. Moreover, in cases II and III, the image formation threshold is the same as 1dB. However, in case IV, due to the concealed scenario, higher artifacts are observed as the EM wave bounces in the box, and hence, a higher threshold of 1.5dB is applied.

Figure 8b–d show the detected objects in cases II, III, and IV, respectively. All the objects (two in case II, four in case III, and one in case IV) are correctly detected, thereby validating the proposed model of object detection for the THz SAR sensing.

## 5. Object Classification

**Figure 9:**
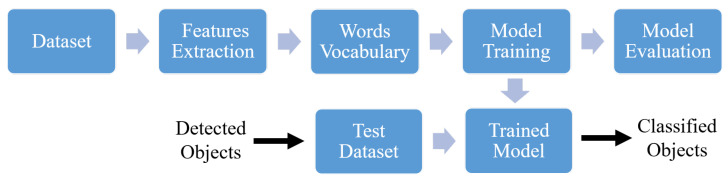
Workflow for object classification.

This section addresses the classification of the detected objects as presented in Figure 8. For classification, a supervised machine learning-based method is employed. The method classifies the detected objects into different classes. Based on the presented cases, the objects can be classified into four classes: “Keyboard”, “Calculator”, “Mobile”, and “USB Stick”. The workflow for object classification is presented in Figure 9. In the following subsections, the modules or blocks of the workflow are described.

### 5.1. Dataset

The dataset for training the model is devised with train–test split approach, where the dataset is categorized into two parts known as the training set and validation set. The train–test slip approach is employed for evaluating the proposed model performance during the training phase. The training set is primarily used for training the model. This set includes the images for which the classifier knows the actual labels during the training phases. On the other hand, the classifier does not know about the labels of the images in the validation set. In this work, 70% of the provided dataset is used for training, and the remaining 30% is used for validation.

Moreover, the collection of the SAR images of the detected objects in cases I–IV serves as the test dataset for final prediction or classification. Generally, the test dataset is different than the dataset for training the model. This condition can only be validated if there is a vast availability of the training data, which is not applicable currently for the emerging THz imaging technology. To the best of the author’s knowledge, there is no public training dataset available for THz images of indoor objects, especially for the considered objects. The SAR images provided in this work are one of the finest images of considered indoor objects. In this work, three schemes are applied to differentiate between the test and training dataset.

Firstly, the SAR raw data are processed only for the 3D space where the object is present. Hence, it results in the SAR image generation of the object instead of the complete case environment. In addition, it is analogous to the measurement performed individually with all the objects. Secondly, only case III is considered for the input data generation as the objects overlap each other in cases I, II, and IV. Lastly, to enrich the input dataset, SAR images of different format and dimensions being small and large are considered. Hence, based on the above three methodologies, the dataset used for training is well distinctive from the test dataset. The dataset is formed of 30 SAR images.

To summarize, the training dataset consists of four classes: keyboard, mouse, mobile, and USB stick, and the classes consist of multiple SAR images of these objects.

### 5.2. Words Vocabulary

Based on the SURF method as explained previously in Section 4, features are extracted, where the dataset is provided as an input. The extracted features are given as input to the word vocabulary module. It forms a bag of words (BoW) or features using the *k*-means clustering algorithm [21]. As a result, *k* different clusters are formed, where features in one cluster resemble each other and differ from those in other clusters. The center of each cluster is a visual word or feature. The BoW model creates a visual dictionary of image features.

### 5.3. Model Training and Evaluation

The output of the BOW creation module is fed to the model training module. The created feature vectors are used to train the classifier. In this work, a supervised machine learning-based Support Vector Machine (SVM) classifier [21] is employed. The classifier categorizes the data based on a best-fit hyperplane. The hyperplane is the decision boundary. Both linear and non-linear boundaries are supported by SVM. Based on the boundaries, the objects are classified into different classes. The features on one side of the boundary differ from the features on the other side of the boundary. For example, in case II, the features of the calculator SAR image would be separated from the features of the USB stick SAR image through the hyperplane. The detailed description of the SVM algorithm is available in [21,32].

The proposed model performance is evaluated with training, validation, and prediction accuracy. The prediction accuracy is also known as model accuracy. The evaluation is based on the correct classification or labeling over total instances. The training and validation accuracy are associated with their respective dataset with the test and split approach, whereas the prediction accuracy is obtained through the test dataset. The goal is to acquire higher accuracy, which defines the correctness of the developed model.

### 5.4. Classified Objects

In the last step of the proposed object classification workflow, the detected object SAR images, which form the test dataset, are provided as input to the trained model. As an output, the respective predicted class or label is obtained. It is worth mentioning that the presented workflow is implemented as an extension of object detection in the same environment (MATLAB), and various available libraries of the above-described algorithms are used.

**Figure 10:**
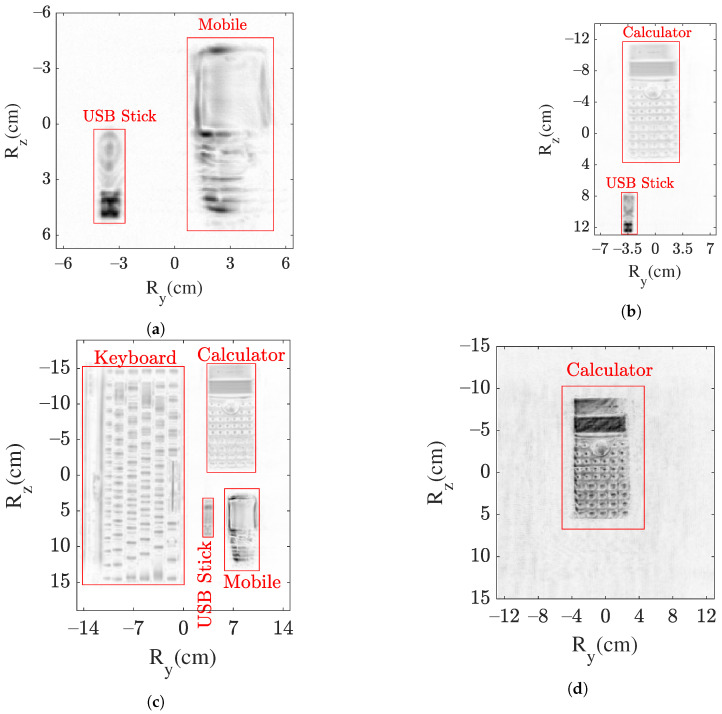
Classification of objects in (**a**) case I, (**b**) case II, (**c**) case III, and (**d**) case IV.

Based on the workflow, the detected objects presented in Figure 8 are provided for classification, and the results are summarized in Figure 10. In cases I–IV, all of the objects are correctly predicted as presented in Figure 10a–d, thus classifying the objects in the THz SAR images. For example, the detected object 2 (shown in Figure 8a) in case I is correctly classified as a USB stick in Figure 10a.

**Figure 11:**
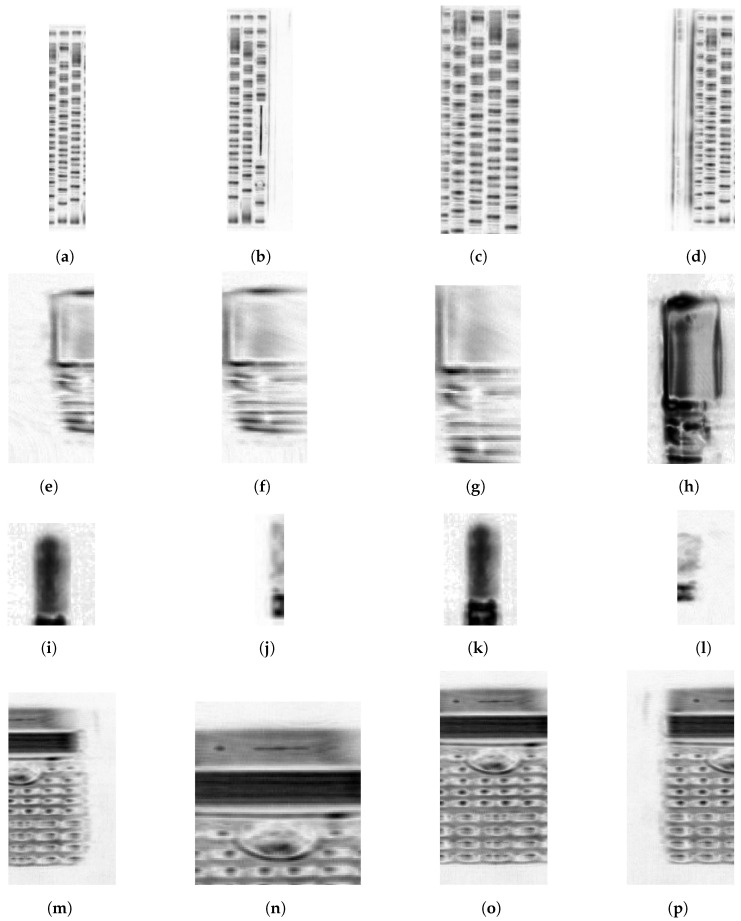
Modified SAR images test dataset with different dimensions and focus plane (**a**–**d**) keyboard, (**e**–**h**) mobile, (**i**–**l**) USB stick, and (**m**–**p**) calculator.

Finally, for the model robustness evaluation, firstly truncated (incomplete) or modified SAR images of the objects are considered. These images are shown in Figure 11, where (a–d), (e–h), (i–l), and (m–p) are the modified images considered in the test dataset of the keyboard, calculator, USB stick, and mobile, respectively. All of these modified images are correctly classified except for Figure 11g,l, which are of a mobile and a USB stick. The addressed robustness evaluation is beneficial as in many cases such as time-critical applications, generating a precise focused SAR image might be complex. In addition, there could be cases that introduce unintentional artifacts. However, based on the proposed method, there is a possibility or opportunity of classifying the object in images with artifacts.

The novelty of the proposed object recognition can be expanded by further evaluating the robustness based on rotated object image and SAR image at a different frequency spectrum, which is accomplished with an imaging technique other than the SAR such as inverse-SAR [5,8]. The principle of inverse-SAR to acquire high angular resolution is similar to SAR. A prime difference is that for inverse-SAR, the movement is performed by the imaging object instead of the transceiver.

**Figure 12:**
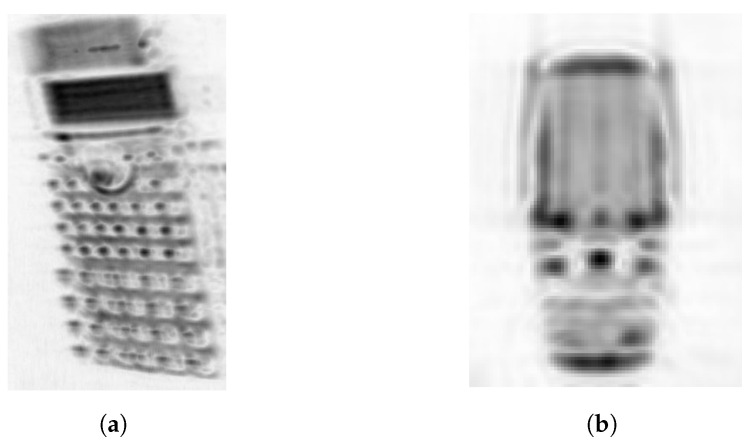
(**a**) Rotated SAR image of a calculator and (**b**) SAR image of the mobile phone in the frequency spectrum of 220–330 GHz.

With regard to the rotated object image, the reconstructed SAR image of a calculator in case II is rotated with random angles along the *y*- and *z*-axis or yaw and pitch directions. The rotated image is presented in Figure 12a. The image is evaluated for classification, and the object is correctly classified. Furthermore, raw data of the mobile phone, mounted on the Y + Z translational stage at a range reference distance of ∼1.2 m, are gathered in a frequency spectrum of 220–330GHz using the inverse-SAR technique. The important measurement parameters can be briefly described as $N_{f}=3001$ and the aperture length along both the *y*- and *z*-axis is 15cm with a step-size of 1mm. The detailed description of the VNA-based measurement setup for the spectrum of 220–330GHz, such as employed antenna dimensions and half power beamwidth, is available in [8]. In reference to Equations (3) and (4), a high-resolution SAR image similar to the spectrum of 325–500GHz can be reconstructed in the selected spectrum of 220–330GHz. Based on the measurement data, the acquired SAR image of the mobile phone in the spectrum of 220–330GHz is shown in Figure 12b. This image is also correctly classified with the implemented model, which is trained with the mobile phone image of the spectrum 325–500 GHz. Hence, it can be summarized that the model is quite robust.

With the test-split approach, both training and validation accuracy of 100% is achieved. Based on the predicted results procured for cases I–IV as presented in Figure 10, the prediction accuracy of 100% is achieved. With the inclusion of the robustness evaluation, the combined prediction accuracy based on results presented in Figure 10, Figure 11 and Figure 12 is ∼93%.

## 6. Conclusions

The presented work focused on acquiring a high-resolution indoor environment map using the THz SAR technique and extended the map with object recognition (detection, localization, and classification). The multi-object indoor environment with four cases including the concealed/hidden object sensing is considered. In addition to object recognition, the evaluation also emphasizes generating a map of a scattered rich environment as the objects are displaced closely. The considered objects are a keyboard, calculator, USB stick, and mobile phone. In all four cases, the objects are well mapped in the frequency spectrum of 325–500GHz. The object in look-through or concealed/hidden object imaging is well mapped, as shown in case IV. The box in case IV provides an attenuation of around −10dB. The acquired SAR map of all the cases is investigated for object recognition.

The proposed model for object detection, localization, and classification is presented and validated. The objects are localized in reference to the transceiver position, and localization accuracy associated with mm spatial resolution is achievable. The proposed workflow for object detection includes image formation, features extraction, and clustering modules. The obtained number of valid clustered groups based on the grouping conditions and group positions provides information on the detected objects. In the four considered cases, all the objects are correctly detected. The detected objects are input to the SVM-based trained model for classification. The developed model performance is evaluated with training, validation, and prediction accuracy. Based on the test-split approach, 70% of the dataset is used for training, and 30% is used for model validation. The trained model achieved the training and validation accuracy of 100%. All the objects in the test dataset based on the considered four cases are correctly classified, and the prediction accuracy of 100% is obtained. Model robustness is also evaluated.

To summarize, the presented results validate the high-resolution environment map generation at the THz spectrum and extension of the map for object recognition, which was primarily dominated by the use of optical spectrum. As an outlook, an enormous training dataset of THz images will be made available publicly, and different machine learning methods will be investigated for comparative analysis.

## Figures and Tables

**Table 1:** Measurement parameters of 3D multi-object imaging.

Symbol	Parameter	Case I	Case II	Case III	Case IV
$f_{c}$	center frequency	412.5GHz	412.5GHz	412.5GHz	412.5GHz
$B_{w}$	bandwidth	175GHz	175GHz	175GHz	175GHz
$R_{\mathrm{ref}}$	reference range	1m	1m	1m	1m
$L_{s,z}$	aperture along *z*-axis	240mm	340mm	284mm	310mm
$L_{s,y}$	aperture along *y*-axis	200mm	150mm	300mm, 270mm	250mm
$\Delta u_{y}$	step size along *y*-axis	1mm	1mm	1mm	1mm
$\Delta u_{z}$	step size along *z*-axis	1mm	1mm	1mm	1mm
$N_{f}$	number of frequency bins	3001	3001	3001	3001
$P_{T}$	base transmit power	−10dBm	−10dBm	−10dBm	−10dBm
-	imaging objects	mobile, USB stick	calculator, USB stick	keyboard, mobile, calculator, USB stick	calculator

## Data Availability

The data presented in this study are available upon reasonable request from the principal author.
